# Vaccination Status of Children With Epilepsy or Cerebral Palsy in Hunan Rural Area and a Relative KAP Survey of Vaccinators

**DOI:** 10.3389/fped.2019.00084

**Published:** 2019-03-26

**Authors:** Li Yang, Jing Peng, Jing Deng, Fang He, Chen Chen, Fei Yin, Shujun Zhang

**Affiliations:** ^1^Department of Pediatrics, Xiangya Hospital, Central South University, Changsha, China; ^2^Xiangya School of Public Health, Central South University, Changsha, China; ^3^Hunan Provincial Center for Disease Control and Prevention, Changsha, China

**Keywords:** China, epilepsy, cerebral palsy, vaccination, KAP survey, vaccinators

## Abstract

**Background:** In China, the vaccination of children with epilepsy (EP) and cerebral palsy (CP) has no specific protocol. Parents are often concerned that vaccination of their children may cause complications due to negative recommendations from vaccinators, resulting in a decline in vaccination. It is therefore is essential to investigate the vaccination status of these specific populations, and the knowledge, attitudes, and practices (KAP) of vaccinators.

**Methods:** This study contains two parts. For the vaccination status survey, residency- and age-matched children whose medical expenditure were covered by the New Rural Cooperative Medical System in Hunan Province were enrolled. Children who were diagnosed with EP or CP were included as the case group, while children without any chronic disease were enrolled as the control group. The vaccination rates of the two groups were compared. For the KAP survey, vaccinators who registered in Hunan CDC were recruited as respondents, and questions were asked related to their experience and attitudes toward vaccinating children with EP or CP.

**Results:** The vaccination rates of the case group were significantly lower than the control group (*P* < 0.001), with the exception of BCG and Hep B_1_. Nine measles and two mumps cases were diagnosed in the case group, while there were no Vaccine Preventable Disease (VPD) cases in the control group. The vaccinators' knowledge of the issues related to the vaccination of children with EP or CP was weaker than their knowledge of general vaccination issues. Furthermore, when making a vaccination decision, seizure-free periods and EEG status were their main concerns.

**Conclusion:** The vaccination status of rural children with EP and CP is in jeopardy in Hunan, China, and there are several misunderstandings regarding the contraindications of vaccines among vaccinators. We suggest that measures are taken to improve this situation.

## Introduction

Since the advent of widespread vaccination programs in the middle of the last century, the global incidence of vaccine-preventable diseases (VPDs) has been significantly reduced. However, a sizeable portion of the population remains more concerned about the adverse effect of some vaccines rather than the risk of VPDs (1, 2). In China, this phenomenon has become particularly severe in the context of children with central nervous system disease. Moreover, these patients often face social stigma and strong psychological burdens (3). The question of whether or not to accept vaccination has been frequently asked in pediatric neurological clinics by parents of children with epilepsy (EP) and cerebral palsy (CP). Many of these parents have reported that their vaccinators often suggest that they forego vaccination for their children to avoid unpredictable complications. However, in China, the incidence of epilepsy is 5 to 10 per thousand per year (4) (which leads to almost 6 million children out of a total population of 9 million that suffer from EP) (5), and the incidence of CP is approximately 1.25 to 6.0 per thousand children (6–9). If this specific population is not appropriately vaccinated, herd vaccination barriers may collapse, leading to disease outbreaks. This is not merely a theoretical concern, as several alarming outbreak incidents have been reported in recent years (10–14).

The arsenal of vaccines available to prevent childhood diseases is quite extensive. Calmette-Guerin vaccine(BCG), Type B Hepatitis vaccine (Hep B), Bacillus Oral Poliomyelitis vaccine (OPV), Diphtheria-Pertussis-Tetanus vaccine (DTP), Measles vaccine/Measles-Mumps-Rubella vaccine (MV/MMR), Meningococcal A vaccine (Men A), Meningococcal A+C vaccine (Men A+C), Japanese Encephalitis vaccine (JEV), and Type A Hepatitis vaccine (Hep A) are required to be inoculated in the general population by the Chinese National Immunization Program (NIP). After years of successful public health system development, Chinese children can now easily get vaccinated at community health service centers. Typically, the vaccinator notifies the parent when a particular vaccination is recommended by the NIP schedule, and the parents decide whether or not to accept, after informed consent is signed. As a primary healthcare protection system operated by the Chinese government, the New Rural Cooperative Medical System (NRCMS) covers 98.7% (802 million) of the rural population in China. All relevant clinical and residency information regarding the diagnostic information and treatments of every patient are recorded in the NRCMS system. Based on the data within the NRCMS, public health researchers can make inquiries on the NIP system as to the vaccination information of each patient. For this reason, children covered by NRCMS could be an ideal model to study the vaccination status of the rural population (15). Hunan province in south-central China is a particularly fitting location for such an investigation, given its 14 subordinate cities and nearly 70 million residents (16). According to the previous report, the prevalence of epilepsy in Hunan fluctuated from 2.8 to 3.7% (17). On the basis of the information obtained from NRCMS for this province, we have investigated the vaccination status of children with EP and CP, while also conducting a KAP survey of vaccine providers (i.e., primary care physicians) within the same population. By comparing the two sets of results, we seek to reveal the difficulties and barriers that may prevent the vaccination of these children, while also proposing some potential solutions.

## Materials and Methods

### Part 1: Vaccination Status and Prevalence of VPDs in Children With EP/CP

#### Inclusion Criteria

Age-matched, 3 to 14 years-old children whose medical expenditures were covered by the NRCMS in Hunan Province were enrolled. Children diagnosed with EP (according to the *International Statistical Classification of Diseases and Related Health Problems 10th Revision*, ICD10-G40) or CP (ICD10-G80) were included as the case group. Children without any chronic disease were enrolled as the control group. The average age of the case group was 4.33 ± 1.82, and the average age of the control group was 4.77 ± 1.56, *P* = 0.221). Four hundred and eighty-seven children with EP or CP were enrolled as the case group, while 500 healthy children were recruited as the control group.

#### Data Sources

The vaccination information for both groups was acquired from the Hunan CDC database of the NIP system. VPD outbreak information was also collected from the Hunan CDC database. Briefly, VPD outbreaks in the patient population were initially reported by the physicians, who collected blood samples. These blood samples were then tested by the subordinate department of infectious disease control for each county or city. Finally, the results of these blood tests were verified by a lab within the Hunan CDC, which is affiliated with the China Infectious Disease Automated-alert and Response System (CIDARS).

#### NIP Vaccine Schedule

Completion age: BCG, Hep B_1_ (the suffix indicates the frequency of administration) within 1 day; MV/MMR, OPV_1−3_, DTP_1−3_, Hep B_2−3_, JEV, and Men A_1_ within 12 months; Men A_2_ within 18 months; Hep A within 24 months; Men A+C within 36 months.

### Part 2: Survey of Health Care Providers Regarding Vaccination Issues of Children With EP/CP

#### Inclusion Criteria

The survey was based on a random-sampling investigation. Vaccinators who registered in the Hunan CDC were included as respondents. All 14 cities affiliated to Hunan Province CDC were involved. Fifty vaccinators registered in each city CDC system were selected randomly by computer to participate. Participants were enrolled in an online survey within a period from June 22 to July 22, 2016 (website: www.web.hnbaili.net).

#### Grouping and Survey Content

The questionnaire was designed by the Department of Pediatrics of Xiangya Hospital and the Xiangya School of Public Health, Central South University. The questionnaire collected the vaccinators' sociodemographic information (gender, age, education, affiliations), and subgroups of the respondents were divided based on age, education, and affiliations. The vaccinators' knowledge, attitudes, and practices regarding the vaccination of children with EP and CP were queried. Questionnaire content is provided in Supplementary Table 1.

#### Quality Control

A preliminary study was conducted. The final questionnaire was followed by the repeated scrutiny and confirmation of pediatric neurologists and epidemiologists from the Central South University.

### Data Analysis

The survey data were collected automatically by the online survey system, while the statistical analysis was run by a second blinded analyst. Descriptive statistics were summarized as percentages. Differences between groups were assessed using chi-square testing. Multiple linear regression was applied to analyze the relationship between sociodemographic variables and the knowledge score of respondents. Logistic regression was applied to examine the relationship between EEG status and the extent of approval for vaccination. Simple linear regression was applied to investigate the relationship between the length of seizure-free periods and the extent of approval for vaccination. A *p* < 0.05 (based on a two-sided test) was considered statistically significant. SPSS Version 22.0 (IBM Corp) was used for statistical analysis.

## Results

### Vaccination Rates

The vaccination information for both the case and control groups are shown in Table 1. The vaccination rates of the case group were significantly lower than the control group for all vaccines except BCG and HepB_1_. The vaccination rates of OPV_1−3_, DTP_1−3_ in the case group ranged from 78.56 to 87.06%, while the control group held a 97.8–99.6% immunization rate for the same vaccines. Vaccination rates for the measles-containing vaccine, meningitis vaccines and Hep A in the case group fluctuated within 34.9–71.46%. While the ratio of vaccination of those vaccines in the control group ranged from 82.4 to 98.6%.

**Table 1:** Vaccination rates for case group vs. control group (%).

**Vaccination rate**	**Case group (*n* = 487) (%)**	**Control group (*n* = 500) (%)**	**χ2**	***P*-value**
BCG	98.36	99.60	3.799	0.051
Hep B1	98.77	99.80	3.371	0.053
Hep B2	97.54	99.60	9.73	0.002
Hep B3	84.60	98.00	56.289	<0.001
OPV1	87.06	100	58.742	<0.001
OPV2	84.60	99.60	80.204	<0.001
OPV3	81.52	99.00	86.663	<0.001
DTP1	86.65	99.40	62.49	<0.001
DTP2	82.96	99.00	78.19	<0.001
DTP3	78.56	97.80	90.388	<0.001
MV/MMR	71.46	98.60	144.196	<0.001
Men A1	69.20	95.00	112.587	<0.001
Men A2	57.29	90.20	133.471	<0.001
Men A+C	34.90	82.40	229.978	<0.001
JEV	67.56	94.60	118.467	<0.001
Hep A	50.92	91.80	202.85	<0.001

*Generally, the vaccination rates of all NIP scheduled vaccines were significantly lower than those observed for the case group. Only BCG and the first dose of Hep B vaccine were exceptions*.

### Prevalence of VPDs

In the case group, nine cases of measles were reported and verified by IgM ELISA assay in a WHO Global Measles and Rubella Laboratory Network accredited lab affiliated with Hunan CDC. Two mumps cases were diagnosed by serum IgM test as well. The ages of the patients in the measles cases were between 3 and 13 years old. In six out of the total of nine cases, the parents stopped vaccination after EP or CP was diagnosed. The other three cases were diagnosed as cerebral palsy after birth and, therefore, did not receive any measles vaccines. Two mumps cases were also identified in a 10 year-old and an 18 year-old, one of whom ceased vaccination after a diagnosis of EP. Fortunately, all of the VPD cases were diagnosed promptly and properly treated, with none suffering severe complications. No VPDs were identified in the control group.

### Survey of Vaccination Providers and Quality Verification

A total of 713 questionnaires were received, with a response rate of 100%. Furthermore, 50 respondents were involved in the on-site verification of the contents of the questionnaire. The consistency rate of the contents on the website and on-site verification was 94%.

### Sociodemographic Information

Age of the respondents: 20–29, 224 individuals (30.3%); 30–39, 306 individuals (43.1%); 40–50, 162 individuals (22.7%); 50+, 28 individuals (3.9%). Education: junior college, 544 individuals (76.3%); bachelor or above, 169 individuals (23.7%). Affiliations: 41 participants (5.8%) were from county hospitals, county CDC or above; the other 672 participants (94.2%) were from community health service stations.

### Knowledge

The accuracy of the providers' responses with regards to general vaccination knowledge was 88–98% (with an average answer accuracy of 95.1%). However, on the issues of vaccination toward specific patients with immune deficiency, EP, or CP, the accuracy of the answers varied much more, ranging from 33.9 to 92.3% (with an average accuracy of 81.9%) (Table 2). The knowledge score of respondents of different age, education background, and affiliation did not differ significantly (*p* > 0.05) (Table 3).

**Table 2:** Immunization knowledge.

**Survey contents (Correct answer)**	**Accuracy rate %**
1. Every vaccine has contraindications (True).	97.1
2. If a patient has vaccine immunization contraindications, he/she cannot receive any vaccine (False).	95.4
3. Most contraindications of vaccines are temporary. When situations such as acute infectious disease recovery or particular physiological condition (such as fever) do not exist, patients can be inoculated (True).	88.2
4. A patient with immune deficiency or whose immune functions are not complete cannot be inoculated with live vaccines but can be inoculated with inactive vaccines (True).	72.8
5. For a patient who has suffered from fever or just recovered from an infectious disease within the past 2 weeks, vaccination can be postponed (True).	96.5
6. For a patient with uncontrolled EP, CP, or encephalitis sequelae, immunization should not include DTP, JEV, or Men A/Men A+C (True).	92.3
7. For a patient with immune deficiency, or whose immune function is inhibited by some medicine, inactivated polio (IPV) can be used instead of OPV (True).	73.8
8. JEV vaccine should not be administered to a patient with fever or infectious disease (True).	98.3
9. A patient who has EP but has maintained a seizure-free status (or who has CP or encephalitis sequela) can receive all kinds of vaccination (True).	33.9
10. EP and CP are contraindications of vaccination, which means that children with these conditions cannot receive any vaccination (False).	70.7

**Table 3:** Multiple linear regression of the relationship between different variables and the knowledge scores of the respondents.

**Coefficients**					
**Model**		**Unstandardized coefficients**		**Standardized coefficients**	***t***	**Significance**
	**B**	**Standard error**	**Beta**			
1	(Constant)	8.236	0.220		37.437	0.000
	Affiliation	−0.169	0.148	−0.031	−1.143	0.253
	Education	0.168	0.098	0.048	1.713	0.087
	Age	−0.029	0.047	−0.018	−0.629	0.529

*Dependent Variable: Score in answering questions about vaccination knowledge*.

*The table shows that neither affiliation, education, nor age had a significant correlation with the vaccination knowledge score of the respondents*.

### Attitudes

The EEG status and the length of the seizure-free periods were the most crucial reference indices of the respondents' attitudes toward vaccination. The approval rate for cases where the patients' EEGs were normal was significantly higher than for cases where the EEGs were abnormal, with an odds ratio of 2.266 (95% CI 1.857–2.766) (Table 4). Additionally, as the length of the seizure-free period was extended, the approval rate for vaccination increased (Table 5). Furthermore, the acceptance rates of the respondents for the measles-containing vaccine (MCV) and pertussis vaccines were < 40% for both children with EP as well as children with CP (Supplemental Table 2). There was no significant difference in the acceptance rate between the respondents of different age, education, and affiliations (*p* > 0.05) (Supplemental Tables 3, 4).

**Table 4:** Attitudes toward vaccination of children with EP under different status conditions (%).

	**Strongly approve**	**Approve**	**DK/REF**	**Against**	**Strongly against**	**Standard error**	**Wald**	**Odds ratio (95% CI)**
Normal EEG^a^	6.6	39.7	10.5	39.7	3.5	0.102	64.808	2.266 (1.857–2.766)
Abnormal EEG^b^	1.1	7.6	7.8	70.7	12.8			

*Parameters: Independent variable*:

a *Assigned Normal EEG to 1*,

b *Assigned Abnormal EEG to 0*;

*Dependent variable: The refusal of the vaccinators was graded on a five-point scale from robust (5) to weak (1). Parameters were analyzed by logistic regression. DK/REF: Don't know/Refuse to answer*.

**Table 5:** Attitudes toward the vaccination of children with EP under different status conditions (%).

	**Strongly approve**	**Approve**	**DK/REF**	**Against**	**Strongly against**	**Standard error**	***t***	**B**	***P*-value**
3 months seizure-free^a^	2.6	13.3	9.4	62.4	12.6	0.094	24.712	2.316	<0.001
12 months seizure-free^b^	3.1	23.8	9.3	56.8	7				
36 months seizure-free^c^	5.9	33.9	8.9	46.45	4.8				

*Parameters: Independent variable*:

a *3 months seizure-free*,

b *12 months seizure-free*,

c *36 months of seizure-free*;

*Dependent variable: The refusal of the vaccinators was graded on a five-point scale from robust (5) to weak (1). Parameters were analyzed by simple linear regression*.

### Practices

**Figure 1:**
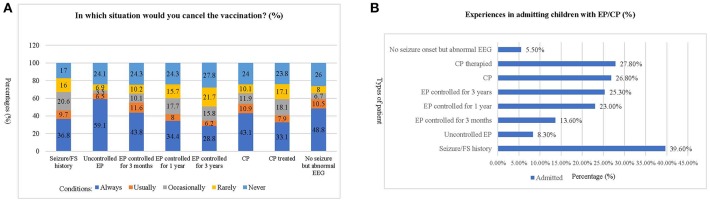
Experiences of respondents in admitting children with epilepsy or cerebral palsy. **(A)** This chart shows the frequency of cancellation for each of the situations described. The most frequent patient type whose vaccination would be canceled by the respondents was “uncontrolled epilepsy,” while the least frequent reason was “epilepsy controlled for 3 years.” **(B)**. This chart shows the percentage of respondents who have experience with different types of EP/CP patients. The most frequent patient type admitted by the respondents was “Seizure/Febrile Seizure history,” which have been encountered by nearly 40% of respondents. In contrast, most of the respondents (nearly 75%) have never admitted the other classes of patient. The least frequently encountered type of patient are those with “no EP onset but abnormal EEG, which only 5.5% (95% CI: 3.8–7.2%) of the respondents have admitted.

The patients whose vaccinations were canceled most frequently by the respondents were those with “uncontrolled epilepsy,” while the cases with the least cancellations were those with “epilepsy controlled for 3 years” (Figure 1A). Two hundred and eighty-two (39.6%) of the respondents reported an experience of admitting patients who had a seizure/febrile seizure history. On the other hand, most respondents (nearly 75%) reported never admitting any of the other classes of patients listed. Only 5.5% of respondents had the experience of admitting patients with no EP onset but with an abnormal EEG (Figure 1B).

**Figure 2:**
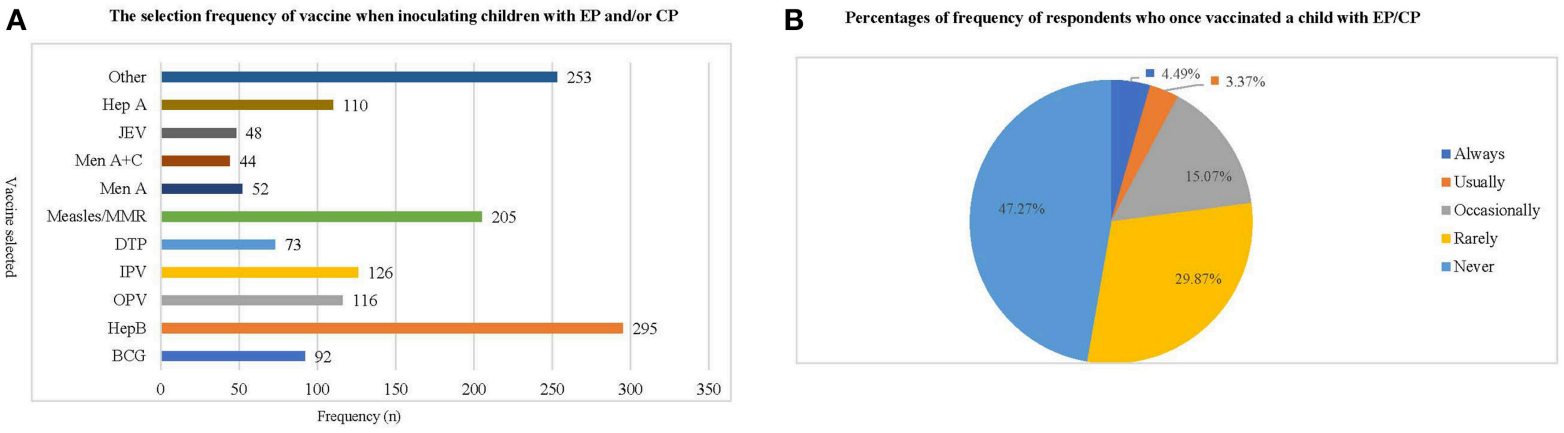
The frequency with which respondents administer vaccinations to children with epilepsy or cerebral palsy. **(A)** This chart shows the frequency with which respondents have administered various types of vaccination to children with EP/CP. Hep B and Measles/MMR vaccine are the inoculations most frequently chosen by respondents, while Men A, Men A+C and JEV are the least frequently chosen. **(B)** This chart shows the percentage of respondents who have ever vaccinated a child with EP/CP, as well as how frequently they have done so. The results indicate that respondents who have never given a vaccination to children with EP and CP accounted for 47.27%, while the choices of “always, usually and occasionally” together accounted for <25% of respondents.

Most respondents (80.9–99.6%) were aware of the importance of avoiding contraindications before vaccination. To ensure this, they asked about each patient's health, immunology, and allergy status before vaccination (Table 6). Hep B and Measles/MMR vaccines were the most preferred choices by the respondents when inoculating patients with EP or CP (Figure 2A). Three hundred and thirty-seven respondents (47.27%) reported never vaccinating, 32 (4.49%) respondents always vaccinate, and 24 respondents (3.37%) usually vaccinate a child with EP and CP (Figure 2B).

**Table 6:** Questions asked before vaccination.

**Questions**	**Percent of respondents who have asked this question (95% CI)**
1. Does your child feel well today? (Physical Examination)	99.6 (99.1–101.1)
2. Does your child have any allergies to food or medicine?	96.5 (95.2–97.8)
3. Did your child have a high fever, convulsions or urticaria after the previous inoculation?	94.0 (92.3–95.7)
4. Does your child have any problem with his/her immune system?	86.5 (84.0–89.0)
5. Has your child received any blood product recently? (Blood or immunoglobulin)	80.9 (78.0–83.8)

*Most respondents (80.9–99.6%) were aware of contraindications before vaccination. The questions they asked were mainly about the subjects' health, immune system, and allergy status*.

The main reasons given for vaccination were “the patient was stable” and “vaccination will be a benefit during the VPD epidemic.” These responses accounted for 63.1 and 54.2%, respectively. On the other hand, the main reason for refusing to vaccinate was “The instructions indicate that EP and CP are contraindications of vaccination,” reported by 80.0% of participants. According to the respondents, the most appropriate way to make a vaccination decision was “The parents decide after they acknowledged the risks and benefits from the doctors,” accounting for 72.7%. Most of the respondents (569, 79.5%) were conscientious to let the parents sign informed consent before vaccination.

### Advice Regarding the Vaccination of Children With EP/CP

The respondents proposed several recommendations. The most common one was “Vaccination should be given after seizures are well-controlled,” accounting for 12.2% (80 individuals). On the topic of how long a patient should be seizure-free before being vaccinated, four (0.6%) respondents suggested 3 months, 14 (2.0%) suggested a year, and eight (11.2%) suggested more than 3 years. Sixty-six (9.3%) of the respondents recommended that patients should go to a specialized hospital for vaccination expertise, and 14 (2.0%) of them recommended that a certification from an expert may be necessary before vaccination was allowed. Forty (5.6%) of the respondents noted that vaccination should follow instructions. Twenty-one (3.1%) of the respondents believed that educating parents about vaccination is crucial. Twelve (1.7%) of the respondents suggested that the government should cooperate with specialists to establish a protocol on vaccination for these children. One respondent proposed that vaccination clinics should be equipped with emergency devices, in case of emergencies after vaccination.

## Discussion

The findings of this research indicates that vaccination rates of children with EP or CP are significantly lower than healthy children, which may put them at greater risk for VPD infection. This result was observed for all vaccines except BCG and Hep B, which are the first vaccines mandatorily given immediately following birth. This phenomenon requires urgent attention, especially from policymakers. Before this study, there were only a few reports about the vaccination status of children who have a neurological disorder in China. In 2003, Lu reported that the vaccination rate of disabled children was only 78.2%; however, that study did not specify which disabled children were studied (18). In 2012, Wu et al. investigated the vaccination rate of children with CP and found an even worse situation: the overall vaccination rate of BCG, OPV, DPT, and MV was only 54.7%. However, the cause of the phenomenon was not thoroughly investigated in that study (19). Even at the global level, the vaccination status of children with neurological diseases has not drawn much attention among public or health professionals, and very few studies have focused on this topic. In a developed country like the United States, Smith et al. found comparable influenza vaccination rates in children with neurologic or neurodevelopmental disorders (20). In Turkey, Dinleyici et al. found that 95.6% of children with neurological diseases were vaccinated according to the routine NIP (21). These results indicated that the vaccination status in developed countries may not be a major issue. However, in Nigeria, Okoro et al. found that 35.2% of children with neurological diseases were not fully vaccinated (22). This indicates that developing countries may have similar situations on this issue.

“One suffering from encephalopathy, uncontrolled epilepsy, or other progressive neurological diseases” is listed in the Chinese pharmacopeia as a contraindication for all NIP vaccines (23). However, in the U.S., the ACIP (Advisory Committee on Immunization Practices) indicated clearly in the 2010 and 2013 guidelines that, for children with CP and controlled EP, only the DTP vaccine is a contraindication when seizure onset occurs 7 days after the first dose and cannot be explained by other reasons. Of note, EP and CP are not contraindications of other vaccines on those guidelines (24). Over half of the respondents of this study reported that they “always” or “usually” cancel the vaccination of children with CP. The most common reason for this refusal was “There are contraindications indicated by the instructions,” noted by 80% of respondents. The confusion on this point may be due to the fact that the pharmacopeia has not clarified the definition of “encephalopathy” in its contraindication (23). In contrast, “encephalopathy” is clearly defined in the ACIP guidelines (24): “coma, consciousness declined, prolonged seizures, etc.” Most of the Chinese vaccinators lack specific knowledge of pediatric neurology, and as a result, cannot fully understand what “encephalopathy” is. Therefore, they tend to err on the safe side by canceling the vaccination in order to avoid risk. The respondents did well in answering general vaccination questions (Question 1–3, Question 5 and Question 8) with an accuracy higher than 90%, but did not perform as well on particular questions about vaccination of children with EP and CP (Question 6, 92.3%, Question 9, 33.9%, and Question 10, 70.7%).

This result indicates that the knowledge of vaccinators in the context of these special patients requires urgent improvement. However, only 12 respondents thought they should be better trained in that aspect, perhaps due to their limited experience in dealing with these specific patients. Nevertheless, fewer than 40% of respondents had the experience to deal with even the most common type of patient-febrile seizure. The respondents showed a great deal of concern about EEG status, and even in cases where the patient in question had been seizure-free for 3 years, only 12.3% of respondents reported being comfortable with vaccinating children with abnormal EEG. One possible explanation might be that they believed vaccination could lead to epileptic discharging and EP onset. However, two large-scale studies on vaccination reactions, conducted by the Institute of Medicine and the Boston Children's hospital, reported that no vaccine could be shown to lead to neurological disease onset or relapse, and that only MMR vaccine might have a relationship with febrile seizure. Moreover, they found no evidence that vaccination would cause epileptic discharging and subsequently result in EP onset (25, 26). On the other hand, while this study was in progress, Chen et al. reported that after news of the “Shandong vaccine event” began spreading on the internet, the willingness of parents to vaccinate their children declined significantly. 74.3% of parents reported concerns about vaccine safety, leading to an approximate 30% decline in vaccination rates (27).

Declines in vaccination rates could lead not only to VPDs, but also to severe complications. Tanabe et al. investigated 58 cases of children with severe myoclonic epilepsy of infancy (SMEI) and found that their overall vaccination rates were <50%. Subsequently, 15 patients in that study were diagnosed with measles, six with mumps, six with rubella, and two with pertussis. Four of the patients suffered severe complications, with two developing severe pneumonia, one becoming unconscious, and one going into a coma (28). These data were consistent with our study, as nine cases (1.85%) of measles were identified in our case-cohort and two cases of mumps (0.41%) were diagnosed. This is a tremendous elevation compared to the incidence of these two diseases in the country as a whole, where the occurrence of measles is around 0.00043%, and for mumps is around 0.018% (29).

The most frequently-selected vaccine by the respondents was Hep B, followed by measles and MMR. The least-selected vaccine was JEV, which had an approval rate of only 6.9%. When asked under what circumstances they would inoculate a child with these two diseases, the most common answers from respondents were “The patient was stable” and “Vaccination will be of benefit during an epidemic.” These responses indicated that most vaccinators are willing to provide vaccination protection to those children as long as there is no risk, or if vaccination is the only choice. Additionally, one respondent proposed that emergency rescuing devices should be placed in every vaccination clinic. This concern reflected that rescuing equipment is lacking in some remote areas, which is consistent with a WHO investigation in 2008 (30).

The vaccination situation of children with EP and CP within Hunan, China is in jeopardy. According to this study, the following recommendations should be considered. First, the Chinese vaccination pharmacopeia must be revised to align with international guidelines, and the contraindications in the pharmacopeia must be clarified in order to offer a practical guide to vaccinators. Second, training of vaccinators on how to deal with exceptional conditions should be initiated. Finally, a new set of guidelines should be established specific to patients with neurological conditions and compiled by pediatric, neurologic, and epidemiologic experts.

## Data Availability

The raw data supporting the conclusions of this manuscript will be made available by the authors, without undue reservation, to any qualified researcher.

## Ethics Statement

This study was approved by the Ethics Review Committee of Central South University and the Hunan CDC. Every participant in the KAP survey provided signed informed consent before taking part in the study.

## Author Contributions

LY performed the majority of the study, collected and analyzed data, wrote the manuscript, and approved the final manuscript as submitted. JD designed the data collection instruments, and coordinated and supervised data collection, critically reviewed the manuscript, and approved the final manuscript as submitted. JP designed part of this study, revised the manuscript critically for important intellectual content, and approved the finalmanuscript as submitted. FH and CC critically revised the manuscript for important intellectual content and approved the final manuscript as submitted. FY conceptualized and designed the study, and revised and approved the final manuscript as submitted. SZ recruited survey respondents, collected some of the data, and approved the final manuscript as submitted. All authors approved the final manuscript as submitted and agree to be accountable for all aspects of the work.

### Conflict of Interest Statement

The authors declare that the research was conducted in the absence of any commercial or financial relationships that could be construed as a potential conflict of interest.
