# Supplementary material for: Vaccination Status of Children With Epilepsy or Cerebral Palsy in Hunan Rural Area and a Relative KAP Survey of Vaccinators
Source: Front Pediatr. 2019 Mar 26;7:84. doi: 10.3389/fped.2019.00084 (PMC6448507; doi:10.3389/fped.2019.00084)
Supplement: Supplementary file 1 [file Table_1.DOCX]

# Supplement Tables 1

# A survey toward vaccinators about vaccination of children with epilepsy(EP) or cerebral palsy(CP)

1. Basic information and practice

1.1 Gender： ① Male ② Female

1.2 Age：

1.3 Affiliation：

1.4 Academic degree：

1.5 When did you start working with vaccinations Y M

1.6 Have you ever admitted children with the disorders underneath and did you cancel or postpone their vaccination?

| No. | Patient status | Admitted  ①Yes ②No | Vaccination Canceled |
| --- | --- | --- | --- |
|  |  |  | ①Always ②Usually ③Occasionally ④Rarely ⑤Never |
| 1.7.1 | Seizure/febrile seizure history |  |  |
| 1.7.2 | Uncontrolled epilepsy |  |  |
| 1.7.3 | Epilepsy controlled for 3 months |  |  |
| 1.7.4 | Epilepsy controlled for 12 months |  |  |
| 1.7.5 | Epilepsy controlled for 36 months |  |  |
| 1.7.6 | Cerebral Palsy |  |  |
| 1.7.7 | Cerebral Palsy treated |  |  |
| 1.7.8 | No epilepsy onset but abnormal EEG |  |  |

2. Knowledge about vaccination

Please choose an answer and mark it with “√.”

| No. | Items | True | False | DK/REF |
| --- | --- | --- | --- | --- |
| 2.1 | Every vaccine has its contradictions. |  |  |  |
| 2.2 | If a patient has a vaccination contraindication, it means he cannot receive any vaccine. |  |  |  |
| 2.3 | Most contraindications of vaccine are temporary. The patient should be inoculated when he/she has no acute infectious disease or special physiological conditions (such as fever, etc.),. |  |  |  |
| 2.4 | A patient with immunol deficiency cannot be inoculated with live vaccines, but he/she can be inoculated with inactive vaccines. |  |  |  |
| 2.5 | Vaccination should be postponed to a patient who has fever or is recovering from any infectious disease within two weeks. |  |  |  |
| 2.6 | Immunization should not include DTP, JEV, and Men A/Men A+C vaccines to a patient with uncontrolled EP, CP or encephalitis sequelae. |  |  |  |
| 2.7 | Inactivated polio (IPV) can be used instead of OPV to a patient with immune deficiency or whose immune function is inhibited by some medicine. |  |  |  |
| 2.8 | JEV should not be administered to a patient with fever or infectious disease. |  |  |  |
| 2.9 | A patient with EP history but who is seizure-free now, or who has CP or encephalitis sequelae can receive all NIP scheduled vaccines. |  |  |  |
| 2.10 | EP and CP are contraindications of vaccination, which means patients with either of two these diseases cannot receive any vaccinations. |  |  |  |

DK/REF: Don’t know/Refuse to answer

**3. Attitudes toward vaccination of children with EP or CP**

Please describe your attitude toward the vaccination of children with EP or CP under specific circumstances as one of the below:

**1 Strongly Approve 2 Approve 3 DK/REF 4 Disagree 5 Strongly Disagree**

| No. | Items | Choice |
| --- | --- | --- |
| 3.1 | Administer all vaccines to children with EP that has been controlled for three months and who have a normal EEG |  |
| 3.2 | Administer all vaccines to children with EP that has been controlled for three months and who have an abnormal EEG |  |
| 3.3 | Administer all vaccines to children with EP that has been controlled for 12 months and who have a normal EEG |  |
| 3.4 | Administer all vaccines to children with EP that has been controlled for 12 months and who have an abnormal EEG |  |
| 3.5 | Administer all vaccines to children with EP that has been controlled for 36 months and who have a normal EEG |  |
| 3.6 | Administer all vaccines to children with EP that has been controlled for 36 months and who have an abnormal EEG |  |
| 3.7 | Administer all vaccines to children with EP that rarely recurs a year after telling parents the pros and cons |  |
| 3.8 | Children with EP should not receive enhanced vaccinations if a seizure occurs within three days after the 1^st^ dose of that vaccine |  |
| 3.9 | Children with EP should not receive vaccinations if any symptoms of encephalopathy happen within seven days after the 1^st^ dose of that vaccine |  |
| 3.10 | Administer pertussis vaccine to children with EP |  |
| 3.11 | Administer measles or MMR vaccine to children with EP |  |
| 3.12 | Administer pertussis vaccine to children with CP |  |
| 3.13 | Administer measles or MMR vaccine to children with CP |  |
| 3.14 | Do not administer whole-cell pertussis vaccine to children with CP but administer acellular pertussis vaccine to them |  |
| 3.15 | Do not administer whole-cell pertussis vaccine to children with EP but administer acellular pertussis vaccine to them |  |
| 3.16 | To reduce fever after pertussis vaccination, let children take acetaminophen or ibuprofen at the same time or within 24 hours of vaccination |  |

4**. Practice and recommendation on vaccination of children with EP or CP**

4.1 Did you ask their parents the questions below before vaccination？

4.1.1 Does your child feel good today? (Physical Examination)（ ） ① Yes ②No

4.1.2 Does your child have any allergic food or medicine? （ ） ① Yes ②No

4.1.3 Did your child have a high fever, convulsions, or urticaria after previous inoculations? （ ） ①Yes ②No

4.1.4 Does your child have any problem with his/her immune system? （ ） ① Yes ②No

4.1.5 Has your child received any blood products recently? （e.g., Blood or immunoglobulin）（ ） ① Yes ②No

4.2 Have you ever inoculated a child with epilepsy or cerebral palsy：（ ）

①Always ②Usually ③Occasionally ④Rarely ⑤Never

*If you answered ①Always, ②Usually, ③Occasionally, or ④Rarely, please answer questions 4.2.1~4.2.3. If not, skip and go ahead to Question 4.2.4.*

4.2.1 If you answered ①Always, ②Usually, ③Occasionally, or ④Rarely，the vaccine you administered was: (Multiple choices)（ ）

①BCG ②Hep B ③OPV ④DTP ⑤MV/MMR ⑥Japanese encephalitis ⑦Group A meningococcal ⑧ Group A+C meningococcal ⑨ Hep A ⑩ Others

4.2.2 Reason for vaccination: (Multiple choices)（ ）

① Improve the vaccination ratio ② Parents’ request ③ The patient was stable

④ Vaccination will be of benefit during an epidemic

4.2.3 Did you require the parents to sign an informed consent before vaccination? （ ）

①Yes ②No

4.2.4 What are the reasons you would refuse to inoculate a child with epilepsy or cerebral palsy? (Multiple choices)（ ）

① **Thinking that they should be vaccinated, but worrying about that vaccination could cause unpredictable complications**

② **The instructions indicate that EP and CP are contraindications of vaccination**

③ **The parents refuse**

4.3 Who and how do you think the vaccinating decision should be made to a child with epilepsy or cerebral palsy?

① Doctors should decide because of the parents lack medical knowledge

② The parents decide after they acknowledged the risks and benefits from the doctors

③ To avoid unnecessary troubles, those children should be handled as the case of contraindications unless their parents insist on vaccination

④ Other (Please explain what and why)

4.4 Have you ever made a recommendation to the parents of children with epilepsy or cerebral palsy on vaccination？（ ） ① Yes ②No

If yes, your recommendation was: (Please describe it briefly)

**Thanks for your cooperation and we wish you all the best in your work!**

**Investigator Reviewer**

**Date and time**
