# Supplementary material for: Vaccination Status of Children With Epilepsy or Cerebral Palsy in Hunan Rural Area and a Relative KAP Survey of Vaccinators
Source: Front Pediatr. 2019 Mar 26;7:84. doi: 10.3389/fped.2019.00084 (PMC6448507; doi:10.3389/fped.2019.00084)
Supplement: Supplementary file 2 [file Table_2.docx]

**Supplemental Table 2: Attitudes toward vaccination of children with EP and CP**

| Survey contents | Strongly approve % (95% CI) | Approve %(95% CI) | DK/REF* % (95% CI) | Disagree %(95% CI) | Strongly  Disagree %  (95% CI) |
| --- | --- | --- | --- | --- | --- |
| 1. Administer all vaccines to children with EP that has been controlled for three months and who have a normal EEG | 4.4(2.9-5.9) | 21.9 (18.9-24.9) | 11.4 (9.1-13.7) | 57.4 (53.8-61.0) | 5.1(3.5-6.7) |
| 2. Administer all vaccines to children with EP that has been controlled for 3 months and who have an abnormal EEG | 0.7 (0.1-1.3) | 4.6 (3.1-5.1) | 7.3 (5.4-9.2) | 67.3 (63.9-70.7) | 20.1 (17.2-23.0) |
| 3. Administer all vaccines to children with EP that has been controlled for 12 months and who have a normal EEG | 5.2 (3.6-6.8) | 40.3 (36.7-43.9) | 11.2 (8.9-13.5) | 40.4 (36.8-44.0) | 3.0 (1.7-4.3) |
| 4. Administer all vaccines to children with EP that has been controlled for 12 months and who have an abnormal EEG | 1.0 (0.3-1.7) | 7.3 (5.4-9.2) | 7.4 (5.5-9.3) | 73.2 (69.9-76.5) | 11.1 (8.8-13.4) |
| 5. Administer all vaccines to children with EP that has been controlled for 36 months and who have a normal EEG | 10.3 (8.1-12.5) | 57.0 (53.4-60.6) | 9.0 (6.9-11.1) | 21.4 (18.4-24.4) | 2.3 (1.2-3.4) |
| 6. Administer all vaccines to children with EP that has been controlled for 36 months and who have an abnormal EEG | 1.5 (0.6-2.4) | 10.8 (8.5-13.1) | 8.8 (6.7-10.9) | 71.5 (68.2-74.8) | 7.3 (5.4-9.2) |
| 7. Administer all vaccines to children with EP that rarely recurs a year after telling parents the pros and cons | 1.7 (0.8-2.6) | 23.0 (19.9-26.1) | 8.3 (6.3-10.3) | 60.0 (56.4-63.6) | 7.0 (5.1-8.9) |
| 8. Children with EP should not receive enhanced vaccination if seizure occurs within 3 days after the 1^st^ dose | 17.3 (14.5-20.1) | 57.9 (54.3-61.5) | 5.9 (4.2-7.6) | 16.6 (13.9-19.3) | 2.4 (1.3-3.5) |
| 9. Children with EP should not receive enhanced vaccination if any symptoms of encephalopathy occurs within 7 days after the 1^st^ dose | 22.8 (19.7-25.9) | 56.9 (53.3-60.5) | 5.8 (4.1-7.5) | 12.1 (9.7-14.5) | 2.5 (1.4-3.6) |
| 10. Administer pertussis vaccine to children with EP | 0.4 (-0.1-0.9) | 7.3 (5.4-9.2) | 10.0 (7.8-12.2) | 77.5 (74.4-80.6) | 4.6 (3.1-6.1) |
| 11. Administer measles or MMR vaccine to children with EP | 1.5 (0.6-2.4) | 31.7 (28.3-35.1) | 16.2 (13.5-18.9) | 45.6 (41.9-49.3) | 4.9 (3.3-6.5) |
| 12. Administer pertussis vaccine to children with CP | 0.6 (0-1.2) | 9.4 (7.3-12.5) | 11.8 (9.4-14.2) | 66.3 (62.8-69.8) | 11.9 (9.5-14.3) |
| 13. Administer measles or MMR vaccine to children with CP | 1.5 (0.6-2.4) | 34.8 (30.7-38.3) | 15.0 (12.4-17.6) | 44.0 (40.4-47.6) | 4.6 (3.1-6.1) |
| 14. Replace whole-cell pertussis vaccine with acellular pertussis vaccine to children with CP | 1.3 (0.5-2.1) | 19.2 (16.3-22.1) | 23.3 (20.2-26.4) | 50.6 (46.9-54.3) | 5.6 (3.9-7.1) |
| 15. Replace whole-cell pertussis vaccine with acellular pertussis vaccine to children with EP | 1.0 (0.3-1.7) | 17.3 (14.5-20.1) | 21.9 (18.9-24.9) | 54.6 (50.9-58.3) | 5.3 (3.7-6.9) |
| 16. To reduce fever after pertussis vaccination, let children take acetaminophen or ibuprofen at the same time or within 24 hours | 1.5 (0.6-2.4) | 8.6 (6.5-10.7) | 12.6 (10.2-15.0) | 62.7 (59.2-66.2) | 14.6 (12.0-17.2) |

*DK/REF: Don’t know/ Refuse to answer.

Supplemental Table 2 showed the period of seizure free, and their EEG status was the most important reference indexes of the respondents’ attitudes toward vaccination. The approval rate for vaccination to those children who were seizure-free for three years and had a normal EEG was the highest. On the other hand, the participants’ acceptance for vaccinate measles-containing vaccine and DPT vaccines to children with EP or CP were less than 40%.
